# The effect of alogliptin on pulmonary function in obese patients with type 2 diabetes inadequately controlled by metformin monotherapy: Erratum

**DOI:** 10.1097/01.md.0000505546.85718.ea

**Published:** 2016-10-21

In the article “The effect of alogliptin on pulmonary function in obese patients with type 2 diabetes inadequately controlled by metformin monotherapy”,^[1]^ which appeared in Volume 95, Issue 33 of *Medicine*, some affiliation information was originally given incorrectly. The article has since been corrected online.
